# Optimizing Nitrogen Balance Is Associated with Better Outcomes in Neurocritically Ill Patients

**DOI:** 10.3390/nu12103137

**Published:** 2020-10-14

**Authors:** Tae Jung Kim, Soo-Hyun Park, Hae-Bong Jeong, Eun Jin Ha, Won Sang Cho, Hyun-Seung Kang, Jeong Eun Kim, Sang-Bae Ko

**Affiliations:** 1 Department of Neurology, Seoul National University Hospital, Seoul 03080, Korea; ttae35@gmail.com (T.J.K.); HBJeong315@gmail.com (H.-B.J.); 2 Department of Critical Care Medicine, Seoul National University Hospital, Seoul 03080, Korea; hejce1001@hanmail.net; 3 Department of Neurology, Inha University Hospital, Incheon 22332, Korea; g2skhome@hanmail.net; 4 Department of Neurosurgery, Seoul National University Hospital, Seoul 03080, Korea; nsdrcho@gmail.com (W.S.C.); hsk4428@yahoo.com (H.-S.K.); eunkim@snu.ac.kr (J.E.K.)

**Keywords:** nitrogen balance, protein, catabolism, critical illness, neurointensive care

## Abstract

Marked protein catabolism is common in critically ill patients. We hypothesized that optimal protein supplementation using nitrogen balance might be associated with better outcomes in the neurointensive care unit (NICU) patients. A total of 175 patients admitted to the NICU between July 2017 and December 2018 were included. Nitrogen balance was measured after NICU admission and measurements were repeated in 77 patients. The outcomes were compared according to initial nitrogen balance results and improvement of nitrogen balance on follow-up measurements. A total of 140 (80.0%) patients had a negative nitrogen balance on initial assessments. The negative balance group had more events of in-hospital mortality and poor functional outcome at three months. In follow-up measurement patients, 39 (50.6%) showed an improvement in nitrogen balance. The improvement group had fewer events of in-hospital mortality (*p* = 0.047) and poor functional outcomes (*p* = 0.046). Moreover, improvement of nitrogen balance was associated with a lower risk of poor functional outcomes (Odds ratio, 0.247; 95% confidence interval, 0.066–0.925, *p* = 0.038). This study demonstrated that a significant proportion of patients in the NICU were under protein hypercatabolism. Moreover, an improvement in protein balance was related to improved outcomes in neurocritically ill patients. Further studies are needed to confirm the relationship between protein balance and outcomes.

## 1. Introduction

Systemic inflammatory responses (SIR), elicited by acute brain injury, may lead to an alteration in metabolic homeostasis [1]. Insufficient caloric intake has been linked to an increase in morbidity and mortality in patients with severe brain damage [2,3,4,5,6]. Several clinical studies reported that there was no significant benefit of protein supplements on ventilator-free days, renal function, and mortality in general critically ill patients [7,8,9]. However, recent studies suggest that protein balance is more important than the total amount of protein or caloric intake in patients in critical conditions [5,6,10,11,12,13]. Protein catabolism, triggered by SIR, may result in the depletion of amino acids essential for cellular or tissue repair and therefore may result in long-term metabolic dysfunction [4,14,15,16]. Despite this evidence, proteins have not been sufficiently supplemented in critically ill patients who require treatment in the neurointensive care unit (NICU) [4,14,15,16,17]. Nutritional protein balance can be simply assessed using nitrogen balance, calculated from protein intake and urinary nitrogen output [18,19]. Although the optimal amount of protein intake in the NICU has not been determined, an individualized nutritional approach to minimize negative protein balance might be beneficial for such patients. Therefore, we aimed to investigate the association between nitrogen balance and clinical outcomes in patients in the NICU.

## 2. Methods

### 2.1. Study Population

We retrospectively identified the patients admitted to the NICU at our institution between July 2017 and December 2018. We excluded patients with (1) age younger than 18 years, (2) duration of NICU stay less than 72 h, and (3) no data on urine urea nitrogen (UUN) measured within 72 h after NICU admission using a 24-h urine collection. We also excluded patients with renal replacement therapy, plasmapheresis, and oliguria (500 mL/day) affecting the result of UUN measurement during study periods. Among the patients who stayed in the NICU for more than seven days, a follow-up UUN measurement was performed. This study was approved by the Institutional Review Board (IRB NO H-1908-072-1054). The need for informed consent was waived by the IRB.

### 2.2. Baseline Characteristics and Clinical Information

Baseline characteristics including age, sex, hypertension, diabetes mellitus, hyperlipidemia, coronary artery disease, and atrial fibrillation; and a history of stroke/transient ischemic attack were evaluated. In addition, we obtained information regarding malignancy and gastrointestinal diseases related to nutritional status. Laboratory evaluations were performed for blood urea nitrogen (BUN) and creatinine at the time of initial and follow up measurements in nitrogen balance. We also assessed the primary diagnosis at NICU admission and the treatment process which might affect energy metabolism (i.e., barbiturate coma therapy, sedation, targeted temperature management [TTM], craniectomy/craniotomy, extraventricular drainage, vasospasm therapy, hyperosmolar therapy, use of anticonvulsant therapy in status epilepticus, and management of sepsis). Moreover, we evaluated the development of sepsis, defined by sepsis-3 criteria [20] or acute respiratory distress syndrome (ARDS) according to the Berlin definition during NICU management [21]. In addition, treatments of transfusion (packed red blood cell, platelets, fresh frozen plasma, etc.) were evaluated in the UUN monitoring periods during NICU hospitalization for evaluating protein contents in each blood product [22,23,24]. The severity of the patient’s condition at admission was assessed using the Acute Physiology and Chronic Health Evaluation (APACHE)-II score [25], and neurological assessments were performed using the Glasgow Coma Scale (GCS) during NICU hospitalization (initial at admission to NICU and follow-up at discharge from NICU).

### 2.3. Nutrition Support with Monitoring

Protein intake (g/kg/day) and caloric intake (kcal/kg/day) were assessed during NICU hospitalization based on the actual measured intake of enteral nutrition (EN), parenteral nutrition (PN), and intravenous fluid including propofol, amino acid, albumin, and blood transfusion. The decision on the route of nutritional supply (EN, PN, or EN with PN) was made in a consultation with a clinical nutritionist considering the patients’ medical condition. Determination of energy requirements including calorie and protein were estimated from the Harris–Benedict equation using a simplistic weight-based value (25–30 kcal/kg/day) and adjusted for disease severity using stress factors [26,27]. Moreover, we calculated nitrogen balance (g/day) using the protein intake and 24-h UUN data (grams of nitrogen excreted in urine over a 24-h period). The 24-h urine for analyzing UUN was obtained from indwelling urinary catheters. The following standard formula was used: total protein intake (g)/6.25 − (UUN + 4 g) [18,19]. Patients were divided into two groups based on the initial nitrogen balance results (positive protein anabolism [nitrogen balance ≥ 0 g/day] or negative protein catabolism [nitrogen balance < 0 g/day]). The nutritional status assessment in the included patients was evaluated using the Nutrition Risk in the Critically Ill (NUTRIC) score [28]. Nutritional support in the included patients was evaluated and received based on the nutritional support team (NST) consultation and intervention. 

A follow-up nitrogen balance measurement was performed among patients who stayed in the NICU for more than seven days. The measurements of follow-up nitrogen balance were performed at approximately seven days after the first examination. Those patients were categorized into two groups based on the results of the follow-up nitrogen balance examination (improvement group; zero or positive nitrogen balance on the follow-up measurement vs. no improvement/aggravation group; persistent negative nitrogen balance or worsened nitrogen balance in comparison to initial nitrogen balance on the follow-up measurement). Cumulative fluid balance was also assessed using input (I) and output (O) during the same period. Body weight, height, body mass index (BMI), and the percentage of ideal body weight (%IBW) were assessed on admission using the IBW formula (IBW, men = 50 kg + 2.3 kg × [height (inches) − 60], women = 45.5 kg + 2.3 kg × [height (inches) − 60]) [29]. In addition, we calculated the %IBW (current body weight/IBW × 100%) to assess the percentage excess or deficit in the IBW. Body weight change was also monitored during the protein monitoring period, and weight loss was defined as a decrease in body weight of more than 3 kg or 5% as compared to initial body weight [30,31]. Those nutritional methods, protein intake, calorie intake, body weight changes, and cumulative fluid balance were evaluated at the time of initial and follow up measurements in nitrogen balance. 

### 2.4. Outcome Assessments

The primary outcome was functional outcome at three months after NICU hospitalization. We assessed functional outcomes using a modified Rankin scale (mRS) score at three months. We divided patients into two groups: those with good outcome (mRS score ≤ 3) and poor outcome (mRS score ≥ 4) [32,33]. Secondary outcomes were in-hospital mortality, neurological worsening defined as an aggravation of ≥2 points compared to the initial in the GCS score [34], ICU or hospital length of stay, and development of acute kidney injury (AKI), which was determined using the Kidney Disease: Improving Global Outcomes (KDIGO) criteria [35]. 

### 2.5. Statistical Analysis

Baseline characteristics are presented as frequency (%) and continuous variables with normal distributions are presented as mean ± standard deviation (SD), while variables that were not normally distributed are presented as median (IQR). Continuous variables were compared using the Student’s t-tests or the Mann–Whitney U-test, and the proportions of categorical variables were compared using the Pearson’s χ^2^ tests or the Fisher’s exact test, as appropriate, to evaluate the relationship between protein balance and outcomes. The association between the improvement of nitrogen balance and outcomes was analyzed using logistic regression analyses. Covariates with statistically significant differences (*P* < 0.05) by univariate analysis based on the one in ten rule and those with clinically important factors were adjusted for multivariable analysis. Moreover, the Kaplan–Meier method was used to compare outcomes and timings between the patients with and without improvement or aggravation of protein balance. For all analyses, a 2-tailed *p*-value < 0.05 was considered statistically significant. Statistical analyses were performed using the SPSS program (Version 25.0, IBM Statistics, Armonk, NY, USA), and GraphPad Prism (Version 8, GraphPad Software, San Diego, CA, USA).

## 3. Results 

In this study, a total of 273 patients were initially enrolled. We excluded patients who were younger than 18 (*n* = 2), stayed less than 72 h in the NICU (*n* = 71), patients without data on urine urea nitrogen (UUN) measured within 72 h after NICU admission (*n* = 9), and those with factors affecting the result of UUN including renal replacement therapy (*n* = 8), plasmapheresis (*n* = 1), and oliguria (500 mL/day) (*n* = 7) were also excluded. Finally, we included 175 patients for analysis. 

Among a total of 175 patients (male, 50.3%; mean age, 59.5 years), 140 patients (80.0%) had a negative nitrogen balance on initial assessment (Table 1). The negative nitrogen balance group was more likely to have lower initial GCS, higher APACHE II score, and higher NUTRIC score. They underwent additional treatments including propofol or barbiturate coma therapy, TTM, decompressive surgery, and other managements including extraventricular drainage, vasospasm therapy, anticonvulsant therapy, and management of sepsis. Regarding nutritional variables, higher protein and calories were supplemented in patients with positive nitrogen balance than in those with negative nitrogen balance (protein: 1.58 ± 0.40 vs. 0.58 ± 0.48 g/kg/day, *P* < 0.001; calories: 25.6 ± 7.3 vs. 11.7 ± 9.5 kcal/kg/day, *p* < 0.001). In addition, EN was a dominant form of nutritional support in the positive balance group, while NPO was more frequent in the negative balance group (*p* = 0.002, Table 1). The time of the first UUN measurement after NICU admission was not different between the two groups (median, 1 (1–2) day vs. 1 (1–2) day, *p* = 0.935). The patients with positive nitrogen balance tended to have good outcomes at three months compared to those with negative protein balance (65.7% vs. 33.6% *p* = 0.001). The percentage of patients with in-hospital mortality (5.7% vs. 20.0%, *p* = 0.045) and neurological worsening (5.7% vs. 23.4%, *p* = 0.015) was significantly higher in the group with negative nitrogen balance. The proportions of poor outcomes such as poor outcome at three months, in-hospital mortality, and neurological worsening were higher in the negative protein balance group according to initial diagnosis (ischemic stroke, subarachnoid hemorrhage, and intracerebral hemorrhage) but there was no statistically significant difference. This might have originated from small sample size numbers of outcome events in each disease (Supplementary Table S1). Moreover, patients with negative nitrogen balance had longer NICU length of stay (13, IQR (7–22) vs. 4, IQR (3–11), *p* < 0.001) and hospital length of stay (28, IQR (18.25–49.5) vs. 19, IQR (12–29), *p* = 0.002) than those with positive nitrogen balance. 

**Figure 1:**
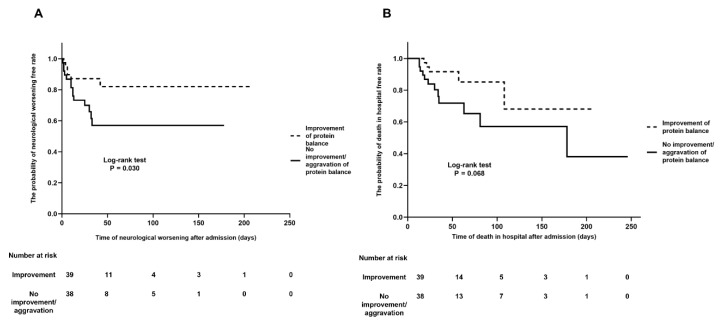
Kaplan–Meier curves for outcomes according to the nitrogen balance. Patients with an improvement in protein balance had fewer events of neurological worsening (*p* = 0.030) (**A**) and lower in-hospital mortality (*p* = 0.068) than those without an improvement or aggravation in protein balance (**B**).

Among included patients (*n* = 175), 77 patients (44.0%) had a follow-up assessment of protein balance on a median of seven days (IQR, 5–8) and 39 patients (50.9%) had an improvement in nitrogen balance compared to initial nitrogen balance. Baseline characteristics and severity of illness on admission were not different between the two groups (Table 2). The median days of initial and follow-up measurements of UUN were not different according to the two groups. In addition, the development of sepsis and ARDS during NICU hospitalization was similar in the two groups. The proportion of subjects with a good functional outcome at three months was significantly greater in the improvement of nitrogen balance group than in the without improvement or aggravation of nitrogen balance group (41.0% vs. 18.4%, *p* = 0.046). Moreover, patients who achieved an improvement in nitrogen balance had lesser neurological worsening (15.4% vs. 36.8%, *p* = 0.032) and lower in-hospital mortality (12.8% vs. 31.6%, *p* = 0.047), even though initial GCS was comparable to those without improvement or aggravation in nitrogen balance (Table 2, Figure 1A,B). The median time of neurological worsening was 10 days (IQR, 3.3–22.5) after NICU admission. Neurological worsening occurred in patients with acute ischemic stroke (*n* = 5) on a median of 10 days after NICU admission due to hemorrhagic transformation in the territory of infarction. In subarachnoid hemorrhage, a neurological worsening (*n* = 3) occurred due to delayed cerebral ischemia on a median of 12 days. In patients with intracerebral hemorrhage, neurological worsening was related to rebleeding and intracranial pressure crisis (*n* = 3, on a median 11 day). Patients with improvement of nitrogen balance had lower odds for poor functional outcome at three months (Odds ratio (OR) 0.247, 95% Confidence Interval (CI) 0.066–0.925, *p* = 0.038). In addition, improvement of nitrogen balance was also associated with lower risk of in-hospital mortality (OR 0.202, 95% CI 0.048–0.858, *p* = 0.030) and neurological worsening (OR 0.177, 95% CI 0.043–0.721, *p* = 0.016) after adjusting for the relevant confounding variables (Table 3).

Patients with an improved nitrogen balance were administered a higher protein amount (1.94 ± 0.63 g/Kg/day vs. 1.28 ± 0.54 g/Kg/day, *p* < 0.001) and higher calories (25.3 ± 7.5 Kcal/Kg/day vs. 21.5 ± 7.9 Kcal/Kg/day, *p* = 0.037) on follow-up than those without improvement or aggravation in nitrogen balance (Table 2). However, initial nitrogen balance, weight change, cumulative fluid balance, and routes of nutritional supplementation during the monitoring period were not different between the two groups. Moreover, the risk of new AKI, defined using KDIGO criteria (7.7% vs. 5.3%, *p* = 1.00, Fisher’s exact test), and level of creatinine were similar and despite the differences in the amount of protein supplementation between the two groups (Table 2). 

## 4. Discussion

In this study, we found that a significant proportion of patients were underfed in terms of nitrogen or protein balance, which was associated with poorer outcomes in the NICU. Moreover, an improvement in nitrogen balance with active nutritional support could be linked to better neurological outcomes. 

A marked increase in protein catabolism has been observed in the acute stage of critical illness, and the types of protein synthesized in the acute phase may differ in patients with severe illness compared to those in healthy conditions, possibly mediated by the intensity of SIR [10,17,36,37]. Conflicting results exist regarding the effect of protein supplementation on outcomes in patients with a critical illness. Several studies have reported that lower protein intake was better in terms of safety, and fewer complications occurred in general critically ill patients [7,8,9,12,13,38,39,40,41,42]. In contrast, other studies have shown that a high protein intake during the acute stage of critical illness is associated with lower mortality and fewer complications and can reduce protein breakdown during the catabolic state [5,12,38,39]. 

However, the optimal methods of protein provision have not been determined in neurologically critically ill patients. Therefore, we investigated whether an optimal protein supplementation, assessed by an improvement in nitrogen balance, was associated with better clinical outcomes in patients with severe neurological illnesses. In our study, an improvement in nitrogen balance (zero to positive balance) from initial nitrogen balance with a higher provision of protein (mean, 1.94 g/kg/day) was associated with better functional outcome, lesser neurological worsening, and lower in-hospital mortality. The amount of protein administered to patients with an improvement in nitrogen balance was close to the recommended amount (2.0–2.5 g/kg/day of protein) from the most recent guidelines and expert opinions [11,17,27,43,44,45,46,47]. High protein supplementation may have adverse events including hypertonic dehydration or development of AKI [48], which was not observed in our patients. 

Protein metabolism is shifted to catabolism due to the effect of SIR in patients with critical illness [17,37,42]. Stress metabolism is a component of the adaptive response to acute illness and is characterized by over-activation of the ubiquitin-proteasome pathway, which causes excessive protein degradation [10,17,36,37,42,49]. In general, a large increase in protein breakdown would lead to an increase in the synthesis of inflammatory mediators. Therefore, a depletion of amino acids essential to the recovery process may lead to an inhibition of protein synthesis and mitochondrial biogenesis and would result in an induction of compensatory energy-recycling responses [47,48,49,50,51]. In contrast, sufficient proteins are activators of anabolic processes that promote cell growth and survival and might promote positive protein balance, decrease inflammation and organ injury, improve immune function, and attenuate tissue damage [51,52,53,54,55,56,57,58,59]. Hypercatabolism and subsequent insufficient nutritional supplementation can rapidly lead to organ dysfunction and an alteration in the appropriate immune response. Therefore, monitoring nitrogen balance is simple but may be crucial in evaluating the status of protein degradation [27,44,46,60]. The optimal protein requirement can be defined as the amount required to maintain a neutral tissue protein balance, at least in physiological conditions [49]. We think that these mechanisms underlying a positive nitrogen balance with optimal protein provision based on serial monitoring may be associated with improvements in neurological outcomes in neurocritically ill patients. 

There were several limitations to our study. First, this is a retrospective study, and a certain degree of unmeasured bias can exist. In addition, the causes of differences in the amount of protein intake were not evaluated. Second, patients with negative nitrogen balance had more severe injuries; thus, a protein underfeeding could be just an epiphenomenon. However, the outcomes were better in patients who showed an improvement in nitrogen balance on follow-up compared to initial nitrogen balance, despite having similar initial severity. Given the nature of the retrospective observational study, we could not conclude the cause and effect relationship. Therefore, special care should be taken in interpreting the results. Third, UUN measurement may underestimate the total amount of protein catabolism in certain conditions. In addition, changes and fluctuations of urea on the day of assessment of UUN was not evaluated. UUN can only measure nitrogen products excreted in the urine; therefore, if patients are suffering from oliguria and renal replacement therapy or have non-renal routes of protein loss through gastrointestinal routes such as massive diarrhea or open abdomen (above 4 g per day), the estimated nitrogen balance may not be accurate [18]. However, the included patients of this study were neurologically ill patients without any conditions of open abdomen or massive diarrhea during monitoring periods, and we excluded patients with oliguria, renal replacement therapy, and plasmapheresis to minimize bias from renal function. Moreover, there was no event related to gastrointestinal diseases in this study. Measuring nitrogen balance based on UUN is a validated and widely accepted method to estimate protein metabolism in several studies [6,7,18,19]. Therefore, we think that UUN measurements played a role in reflecting the state of protein catabolism in our patients. Fourth, we could not measure caloric expenditure using more accurate indirect calorimetry, which could have affected the outcomes [11,36,49]. Fifth, micronutrients, such as trace elements and vitamins, were not considered in our study [27,45,46]. Sixth, patients with less severity had a shorter ICU stay, a follow-up nitrogen balance was measured in relatively more severe patients. Therefore, the association between improvement of nitrogen balance and clinical outcome should be interpreted with caution. Seventh, an improvement in generalized edema could lead to body weight loss, which is not the case in this study. Although we could not directly measure the degree of generalized edema, most patients had a positive fluid balance. Therefore, we do not think that the body weight loss was simply due to an improvement in generalized edema. Despite these limitations, however, we think that our study presents a valid correlation between improving protein balance through adequate protein provision and short-term outcomes in neurocritically ill patients.

## 5. Conclusions

In conclusion, this study demonstrated that improving nitrogen balance was related to better functional outcomes and lower in-hospital mortality in NICU patients. Whether catabolism and nitrogen losses can be reversed by enhanced individualized nutritional support, and whether this improves outcome, remains to be investigated in neurologically ill patients. Furthermore, this result needs to be verified by a randomized study using a larger number of patients.

## Figures and Tables

**Table 1:** Clinical characteristics according to initial nitrogen balance.

	Positive Nitrogen Balance (*n* = 35, 20%)	Negative Nitrogen Balance (*n* = 140, 80%)	*p*-Value
Age (years), mean ± SD	56.4 ± 15.7	60.3 ± 18.1	0.246
Male, *n* (%)	13 (37.1)	75 (53.6)	0.082
BMI (kg/m^2^), mean ± SD	22.5 ± 3.8	23.0 ± 4.0	0.478
HT, *n* (%)	14 (40.0)	60 (42.9)	0.760
DM, *n* (%)	4 (11.4)	32 (22.9)	0.135
HL, *n* (%)	5 (14.3)	21 (15.0)	0.915
CAD, *n* (%)	0 (0.0)	9 (6.4)	0.207
A. fib, *n* (%)	1 (2.9)	17 (12.1)	0.129
Previous stroke/TIA, *n* (%)	4 (11.4)	22 (15.7)	0.607
Cancer, *n* (%)	2 (5.7)	17 (12.1)	0.372
GI diseases, *n* (%)	1 (2.9)	4 (2.9)	1.000
Initial GCS, median (IQR)	13 (9–14)	8 (6–12.75)	<0.001
F/U GCS, median (IQR)	14 (12–15)	11 (5–13)	<0.001
APACHE II score, median (IQR)	21 (15–24)	24 (19–28)	0.025
NUTRIC score, median (IQR)	3 (2–4)	4 (3–5)	0.006
Protein balance * (g/day), mean ± SD,	20.2 ± 23.5	−61.4 ± 39.6	<0.001
% of IBW, mean ± SD	107.4 ± 20.1	109.4 ± 20.4	0.614
Protein (g/kg/day), mean ± SD	1.58 ± 0.40	0.58 ± 0.48	<0.001
Calories (kcal/kg/day), mean ± SD	25.6 ± 7.3	11.7 ± 9.5	<0.001
Diagnosis, *n* (%)			0.056
IS	1 (2.9)	25 (17.9)	
SAH	9 (25.7)	21 (15.0)	
SDH	2 (5.7)	22 (15.7)	
ICH	10 (28.6)	23 (16.4)	
SE	2 (5.7)	5 (3.6)	
Others	11 (31.4)	44 (31.4)	
Nutrition supplement, *n* (%)			0.002
NPO	0 (0)	32 (23.0)	
EN	32 (94.1)	101 (75.9)	
PN	2 (5.9)	6 (4.3)	
Managements, *n* (%)			<0.001
Coma therapy	0 (0)	8 (5.7)	
TTM	0 (0)	8 (5.7)	
Decompressive surgery	0 (0)	10 (7.1)	
Decompressive surgery with TTM	0 (0.0)	6 (4.3)	
Others	2 (5.7)	35 (25.0)	
Blood transfusion, *n* (%)			1.000
Packed red blood cell	0 (0.0)	3 (2.1)	
Platelets	0 (0.0)	1 (0.7)	
NICU admission to UUN day, median (IQR)	1 (1–2)	1 (1–2)	0.935
Good outcome at 3 months (mRS = 0–3), *n* (%)	23 (65.7)	47 (33.6)	0.001
Neurological worsening, *n* (%)	2 (5.7)	34 (24.3)	0.015
In hospital mortality, *n* (%)	2 (5.7)	29 (20.7)	0.038
Hospital length of stay, median (IQR)	19.0 (12.0–29.0)	28.0 (18.25–49.50)	0.002
NICU length of stay, median (IQR)	4.0 (3.0–11.0)	13.0 (7.0–22.0)	<0.001

* Protein balance = nitrogen balance × 6.25 (g/day); BMI: body mass index, HT: hypertension, DM: diabetes mellitus, HL: hyperlipidemia, CAD: coronary artery disease, A. fib: atrial fibrillation, TIA: transient ischemic attack, GI: gastrointestinal, GCS: Glasgow Coma Scale, IQR: interquartile range, F/U: follow up, IBW: ideal body weight, IS: ischemic stroke, SAH: subarachnoid hemorrhage, SDH: subdural hemorrhage, ICH: intracerebral hemorrhage, SE: status epilepticus, NPO: nil per os, EN: enteral nutrition, PN: parenteral nutrition, TTM: targeted temperature management, UUN: urine urea nitrogen, NICU: neurointensive care unit, mRS: modified Rankin Scale.

**Table 2:** Clinical characteristics according to the improvement of nitrogen balance.

	Improvement of Nitrogen Balance (*n* = 39, 50.6%)	No Improvement/Aggravation of Nitrogen Balance (*n* = 38, 49.4%)	*p*-Value
Age (years), mean ± SD	62.7 ± 15.1	58.6 ± 17.5	0.273
Male, *n* (%)	21 (53.8)	20 (52.6)	0.915
BMI (Kg/m^2^), mean ± SD	22.1 ± 4.2	23.0 ± 3.9	0.375
HT, *n* (%)	16 (41.0)	19 (50.0)	0.429
DM, *n* (%)	7 (17.9)	11 (28.9)	0.254
HL, *n* (%)	5 (12.8)	5 (13.2)	1.000
CAD, *n* (%)	3 (7.7)	2 (5.3)	1.000
A. fib, *n* (%)	5 (12.8)	5 (13.2)	1.000
Previous stroke/TIA, *n* (%)	12 (30.8)	3 (7.9)	0.011
Cancer, *n* (%)	3 (7.7)	5 (13.2)	0.481
GI diseases, *n* (%)	1 (2.6)	1 (2.6)	1.000
Initial GCS, median (IQR)	6.0 (5–11)	6.5 (5–11.25)	0.750
F/U GCS, median (IQR)	9.0 (6.0–13.0)	7.0 (3–12.25)	0.075
APACHE II score, median (IQR)	25 (21–28)	26 (21.5–28.5)	0.478
NUTRIC score, median (IQR)	4 (3–5)	4 (3–5)	0.343
Nutritional support, *n* (%)	39 (97.4)	34 (89.5)	0.200
Initial protein balance (g/day), mean ± SD	−58.3 ± 40.6	−55.7 ± 40.7	0.782
Initial negative nitrogen balance, *n* (%)	36 (92.3)	35 (92.1)	1.000
% of IBW, mean ± SD	105.7 ± 21.3	110.8 ± 21.1	0.289
Body weight loss, *n* (%)	17 (43.6)	19 (50.0)	0.573
Total I/O, mean ± SD	1742.3 ± 3630.6	1658.8 ± 2384.7	0.906
Negative I/O, *n* (%)	12 (30.8)	9 (23.7)	0.485
Diagnosis, *n* (%)			0.445
IS	10 (25.6)	5 (13.2)	
SAH	10 (25.6)	8 (21.1)	
SDH	6 (15.4)	8 (21.1)	
ICH	6 (15.4)	8 (21.1)	
SE	2 (5.1)	1 (2.6)	
Others	5 (12.8)	11 (28.9)	
Initial Nutrition supplement, *n* (%)			0.077
NPO	8 (20.5)	13 (34.2)	
EN	27 (69.2)	25 (65.8)	
PN	4 (10.3)	0 (0.0)	
F/U Nutrition supplement, *n* (%)			0.341
EN	17 (43.6)	23 (60.5)	
PN	3 (7.7)	3 (7.9)	
EN with PN	19 (48.7)	12 (31.6)	
Protein intake on admission (g/kg), mean ± SD	0.66 ± 0.56	0.54 ± 0.48	0.289
Calorie intake on admission (kcal/kg), mean ± SD	12.0 ± 10.1	10.4 ± 9.00	0.455
Protein intake on follow up (g/kg), mean ± SD	1.94 ± 0.63	1.28 ± 0.54	<0.001
Calorie intake on follow up (kcal/kg), mean ± SD	25.3 ± 7.5	21.5 ± 7.9	0.037
Initial BUN (mg/dl), mean ± SD	16.3 ± 8.3	16.0 ± 10.1	0.885
F/U BUN (mg/dl), mean ± SD	23.9 ± 9.6	22.9 ± 11.8	0.692
Initial Creatinine (mg/dl), mean ± SD	0.64 ± 0.30	0.71 ± 0.43	0.389
F/U Creatinine (mg/dl), mean ± SD	0.62 ± 0.39	0.68 ± 0.49	0.551
Event, *n* (%)			0.348
Coma therapy	2 (5.1)	3 (7.9)	
TTM	2 (5.1)	3 (7.9)	
Decompressive surgery	6 (15.4)	2 (5.3)	
Decompressive surgery with TTM	1 (2.6)	5 (13.2)	
Others	2 (5.1)	3 (7.9)	
Blood transfusion, *n* (%)			0.115
Packed red blood cell	0 (0.0)	3 (7.9)	
Sepsis during ICU care, *n* (%)	5 (12.8)	3 (7.9)	0.711
ARDS during ICU care, *n* (%)	5 (12.8)	2 (5.3)	0.431
Development of AKI, *n* (%)	3 (7.7)	2 (5.3)	1.000
NICU admission to initial UUN day, median (IQR)	1 (1–2.3)	1.5 (1–2)	0.805
NICU admission to F/U UUN day, median (IQR)	6 (5–9)	7 (4–8)	0.692
Good outcome at 3 months (mRS = 0–3), *n* (%)	16 (41.0)	7 (18.4)	0.046
Neurological worsening, *n* (%)	6 (15.4)	14 (36.8)	0.032
In-hospital mortality, *n* (%)	5 (12.8)	12 (31.6)	0.047
Hospital length of stay (days), median (IQR)	38.0 (25.0–75.0)	33.5 (23.0–67.25)	0.610
NICU length of stay (days), median (IQR)	18.0 (10.0–32.0)	20.50 (13.75–33.25)	0.815

BMI: body mass index, HT: hypertension, DM: diabetes mellitus, HL: hyperlipidemia, CAD: coronary artery disease, A. fib: atrial fibrillation, TIA: transient ischemic attack, GI: gastrointestinal, GCS: Glasgow Coma Scale, F/U: follow up, IBW: ideal body weight, I/O: input/output, IS: ischemic stroke, SAH: subarachnoid hemorrhage, SDH: subdural hemorrhage, ICH: intracerebral hemorrhage, SE: status epilepticus, NPO: nil per os, EN: enteral nutrition, PN: parenteral nutrition, BUN: blood urea nitrogen, ARDS: acute respiratory distress syndrome, AKI: acute kidney injury, mRS: modified Rankin Scale, TTM: targeted temperature management, UUN: urine urea nitrogen, IQR: interquartile range, NICU: neurointensive care unit.

**Table 3:** Multivariable analyses of the relationship between the improvement of nitrogen balance and outcomes in patients with follow-up nitrogen balance measurements.

	Crude Odds Ratio	95% CI	*p*-Value	Adjusted Odds Ratio	95% CI	*p*-Value
Poor outcome at three months						
Age	1.014	0.984–1.044	0.374	1.007	0.958–1.059	0.793
Initial GCS	0.871	0.761–0.996	0.044	0.825	0.696–0.977	0.026
Initial positive nitrogen balance	0.392	0.073–2.108	0.275	0.314	0.042–2.369	0.261
Improvement of nitrogen balance	0.325	0.115–0.918	0.034	0.247	0.066–0.925	0.038
In-hospital mortality						
Age	1.011	0.977–1.047	0.524	1.048	0.986–1.113	0.134
Initial GCS	1.088	0.942–1.256	0.252	1.086	0.913–1.292	0.350
Initial positive nitrogen balance	0.688	0.075–6.318	0.741	0.565	0.053–5.999	0.636
Improvement of nitrogen balance	0.319	0.100–1.018	0.054	0.202	0.048–0.858	0.030
Neurological worsening						
Age	1.000	0.970–1.032	0.978	1.027	0.975–1.082	0.314
Initial GCS	1.073	0.936–1.230	0.310	1.076	0.911–1.269	0.389
Initial positive nitrogen balance	0.547	0.060–4.992	0.593	0.459	0.042–4.966	0.522
Improvement of nitrogen balance	0.312	0.105–0.928	0.036	0.177	0.043–0.721	0.016

GCS: Glasgow Coma Scale. Adjusting for age, initial GCS, previous stroke, diabetes mellitus, atrial fibrillation, cancer, APACHE II, NUTRIC, sepsis, acute respiratory distress syndrome, initial nitrogen balance, and improvement of nitrogen balance.

## Data Availability

Data supporting the findings of this study are available from the corresponding author (Sang-Bae Ko) on reasonable request.

## Supplementary Materials

The following are available online at https://www.mdpi.com/2072-6643/12/10/3137/s1, Supplementary Table S1: Relationship between nitrogen balance and outcomes according to initial diagnosis.
