# Supplementary material for: Optimizing Nitrogen Balance Is Associated with Better Outcomes in Neurocritically Ill Patients
Source: Nutrients. 2020 Oct 14;12(10):3137. doi: 10.3390/nu12103137 (PMC7602201; doi:10.3390/nu12103137)
Supplement: Supplementary file 1 [file nutrients-12-03137-s001.pdf]

Supplementary Table S1. Relationship between nitrogen balance and outcomes according to initial diagnosis

	Positive nitrogen balance, n (%)	Negative nitrogen balance, n (%)	P-value
**IS (n = 26, 14.9%)**	1 (3.8)	25 (96.2)	
**Good outcome at 3 months (mRS = 0-3), n (%)**	1 (100)	10 (40.0)	0.423
**Neurological worsening, n (%)**	0 (0.0)	9 (36.0)	1.00
**In hospital mortality, n (%)**	0 (0.0)	8 (32.0)	1.000
**SAH (n = 30, 17.1%)**	9 (30.0)	21 (70.0)	
**Good outcome at 3 months (mRS = 0-3), n (%)**	6 (66.7)	11 (52.4)	0.691
**Neurological worsening, n (%)**	1 (11.1)	3 (14.3)	1.000
**In hospital mortality, n (%)**	1 (11.1)	3 (14.3)	1.000
**ICH (n = 33, 18.9%)**	2 (8.3)	22 (91.7)	
**Good outcome at 3 months (mRS = 0-3), n (%)**	2 (100.0)	7 (31.8)	0.130
**Neurological worsening, n (%)**	0 (0.0)	3 (13.6)	1.000
**In hospital mortality, n (%)**	0 (0.0)	3 (13.6)	1.000

IS: ischemic stroke, mRS: modified Rankin Scale, SAH: subarachnoid hemorrhage, ICH: intracerebral hemorrhage
